# Commentary: *Amhr2*-Cre-Mediated Global *Tspo* Knockout

**DOI:** 10.3389/fendo.2020.00472

**Published:** 2020-07-24

**Authors:** Vimal Selvaraj, Kanako Morohaku, Prasanthi P. Koganti, Jianmin Zhang, Wei He, Susan M. Quirk, Douglas M. Stocco

**Affiliations:** ^1^Department of Animal Science, College of Agriculture and Life Sciences, Cornell University, Ithaca, NY, United States; ^2^Division of Animal Science, School of Science and Technology, Institute of Agriculture, Shinshu University, Nagano, Japan; ^3^State Key Laboratory of Medical Molecular Biology, Department of Immunology, Research Center on Pediatric Development Diseases, Institute of Basic Medical Sciences, Chinese Academy of Medical Sciences and School of Basic Medicine, Peking Union Medical College, Beijing, China; ^4^Department of Cell Biology and Biochemistry, Texas Tech University Health Sciences Center, Lubbock, TX, United States

**Keywords:** ovary, testis, theca cells, granulosa cells, Sertoli cells, Leydig cells, steroidogenesis, mitochondria

We have carefully read the recent article by Fan et al. that *Amhr2*^*cre*/+^-mediated deletion produces “global” knockouts rather than tissue specific conditional knockouts (1). Such a major observation has not been explicitly recorded in the >100 publications that demonstrate tissue-specific conditional deletions using this *Amhr2*^*tm*3(*cre*)*Bhr*^ mouse [MGI ID: 3042214; noted previously in (2)]. Furthermore, the authors directed their findings toward questioning published reports which disprove a role for the mitochondrial translocator protein (TSPO) in cholesterol transport and steroidogenesis, one of which was based on *Amhr2*^*cre*/+^-mediated conditional *Tspo* knockout mice (3).

## Transmission From the Male or Female Parent Matters in *Amhr2-Cre* Activity

For cell type specific deletions using *Amhr2*^*cre*/+^ expressing mice (4), it is important to use male *Amhr2*^*cre*/+^ mice in the breeding scheme. This is information that has been shared among colleagues who have previously used *Amhr2*^*cre*/+^ mice in their studies. Accordingly, we have used males to transmit *Amhr2*^*cre*/+^ with confirmed conditional recombination for floxed targets (3, 5–9). Separate from our TSPO studies, there have indeed been incidents with breeding of female *Amhr2*^*cre*/+^ mice that resulted in global deletions. For example: when both male and female *Amhr2*^*cre*/+^ mice were used to delete *Smo*, global knockouts of which are embryonic lethal (10), *Amhr2*^*cre*/+^ conditional knockouts were obtained only when the sire harbored *cre* (unpublished). The breeding scheme was subsequently restricted to using male *Amhr2*^*cre*/+^ mice (8). This is consistent with male-restricted breeding schemes indicated by many different groups (11–21). So, we agree with findings reported by Fan et al. regarding global deletions (1), but caution that this is the case only when female *Amhr2*^*cre*/+^ mice are used [gender of *Amhr2*^*cre*/+^ mice used in his publication was not indicated (1)]. This report therefore does not necessitate reinterpretation of any previous data on *Amhr2*^*cre*/+^ conditional knockouts unless female *Amhr2*^*cre*/+^ mice were inadvertently used.

For some transgenic lines, it is known that *cre* activity can vary based on whether the transgene is inherited from the male or female parent. As a knock-in *Amhr2*^*cre*/+^ allele, expression is anticipated to reflect endogenous *Amhr2* expression; therefore, this aberration is indeed unexpected. As *Amhr2* knockout mice are viable (22), there appears to be little impact if there is indeed endogenous *Amhr2* expression in the inner cell mass and trophoblast layer. It would also be valuable to test if this phenotype can be reproduced with the newly available *Amhr2*^*em*1(*cre*)*Smoc*^ knock-in mice (NM-KI-190012; Shanghai Model Organisms).

## TSPO Is Not Involved in *de novo* Steroidogenesis

Irrespective of method used or the timing of recombination, the conclusion for TSPO-loss of function with regard to *de novo* steroidogenesis is quite consistent [reviewed in (23)]. It does not affect viability (whole animal) nor does it affect mitochondrial cholesterol import in phenotypes reported by several independent laboratories (including ours) that have generated global *Tspo* knockouts using *Ddx4*-cre (24), *Pgk1*-cre (25), *Prm*-cre (26), *Camk2a*-cre (27), and *Best1*-cre (28). The important points are: (a) Four of the above global knockouts generated were using independently produced *Tspo* floxed mice (24–27). (b) One of the global knockouts (28), was generated using the same *Tspo* floxed mouse used by the Papadopoulos group that includes this study by Fan et al. (1). (c) All the global knockouts were derived by recombination of *Tspo*-floxed alleles in the germline [expected (24, 26) or aberrant (27, 28)] or in early diploid cells (25). Therefore, these studies do describe *Tspo* knockouts that lack expression in the inner cell mass, as described for the *Amhr2*^*cre*/+^ -mediated *Tspo* deletion by Fan et al. (1), and reveal no effects on embryonic development. (d) The proposed mechanism of TSPO function in steroidogenesis is by mediating mitochondrial cholesterol import, without which *de novo* steroidogenesis cannot occur [reviewed in (29)]. However, “subtle steroidogenic abnormalities” indicated in post-pregnenolone intermediates observed with TSPO loss in one study (27), is not associated with mitochondrial cholesterol import, but indicative of systemic perturbations across all TSPO expressing tissues involved in energy metabolism (30), and could certainly include effects mediated by the pituitary (31). It must be noted that high TSPO expression is not restricted to steroidogenic cells (32), and the question has always been whether it impacts *de novo* steroidogenesis supporting its conjectural function first proposed in the late 1980s [reviewed in (33)].

Compared to TSPO studies that refute a role in *de novo* steroidogenesis (3, 24–26, 32, 34, 35), there is no straightforward path to explaining the recent contradictory reports made by the Papadopoulos group using mice (1, 36), rats (37), and cells (38) that support their long-promulgated view of TSPO function in mitochondrial cholesterol import. We have previously pointed out these concerns (2, 39), and new evidence from independent groups continue to disassociate anticipated TSPO effects in astrocytes (40), microglia (35), and retinal pigment epithelial cells (41). In reference to this recent manuscript by Fan et al. (1), they report a ~50% decrease in circulating testosterone while in their previous study using mice from the same background and with confirmed Leydig cell *Tspo* deletions, they reported no effect on circulating testosterone levels (36). Despite lower testosterone, there was no evidence of hypogonadism; in fact, testis size significantly increased in their *Amhr2*^*cre*/+^ *Tspo* knockout mice (1). Furthermore, this study by Fan et al. used RNA-seq datasets that we generated for the *Ddx4*-cre *Tspo*-knockout adrenal (24) and *Prm*-cre *Tspo*-knockout lung (26), to suggest that there is some sort of steroidogenic compensation to *Tspo* deletion, when we found no such links in our respective original studies. These RNA-seq datasets show that a compensation to mitochondrial cholesterol import does not exist as the major players implicated in this process were unchanged. Nonetheless, Fan et al. seem to shift context toward explaining TSPO-associated effects on mitochondrial membrane potential and metabolism (1), distinct from the asserted cholesterol binding-transport function (33). Although logical, these elucidations are unrelated and do not justify that TSPO loss-of-function is linked to the first step in steroid biosynthesis as opined throughout the text. There is no loss to mitochondrial membrane potential in hepatocytes (42), or Leydig cells (32), associated with TSPO deletion as suggested (1). Regardless, TSPO is clearly poised to affect cellular function and physiology. Based on *Tspo* knockout studies, this is via a primary function without impact on *de novo* steroid biosynthesis.

## Concluding Remarks

This manuscript obviously represents a commendable effort by Papadopoulos and colleagues to seek evidence linking TSPO and mitochondrial cholesterol import for steroidogenesis. Nonetheless, previous demonstrations that TSPO is not involved in *de novo* steroidogenesis remain substantive and justified. Moving forward, we believe it is important to first acknowledge that high TSPO expression is not only seen in steroidogenic cells; a unifying investigation into mechanism of action across different cell types will be necessary to seek a functional designation for this protein.

## Author Contributions

VS wrote the first draft. KM, PK, JZ, WH, SQ, and DS made edits and revisions. All authors have made substantial intellectual contribution to the work and approved it for publication.

## Conflict of Interest

The authors declare that the research was conducted in the absence of any commercial or financial relationships that could be construed as a potential conflict of interest.
